# Dual PPARα/γ agonist aleglitazar confers stroke protection in a model of mild focal brain ischemia in mice

**DOI:** 10.1007/s00109-019-01801-0

**Published:** 2019-05-30

**Authors:** Valérie Boujon, Ria Uhlemann, Stephanie Wegner, Matthew B. Wright, Ulrich Laufs, Matthias Endres, Golo Kronenberg, Karen Gertz

**Affiliations:** 1 Charité – Universitätsmedizin Berlin, Freie Universität Berlin, Humboldt-Universität zu Berlin, Berlin Institute of Health, Klinik und Hochschulambulanz für Neurologie und Centrum für Schlaganfallforschung Berlin (CSB), Charité Campus Mitte, Charitéplatz 1, 10117 Berlin, Germany; 2 Present Address: pRED, Pharma Research & Early Development, F. Hoffmann-La Roche AG, Strekin AG, Basel, Switzerland; 3 Klinik und Poliklinik für Kardiologie, Universitätsklinikum Leipzig, 04103 Leipzig, Germany; 4 DZHK (German Center for Cardiovascular Research), 10115 Berlin, Germany; 5 Deutsches Zentrum für Neurodegenerative Erkrankungen (DZNE), 10117 Berlin, Germany; 6 College of Life Sciences, University of Leicester, and Leicestershire Partnership NHS Trust, Leicester, UK

**Keywords:** Aleglitazar, Microglia, PPARα, PPARγ, Neuroinflammation, Stroke

## Abstract

**Abstract:**

Peroxisome proliferator-activated receptors (PPARs) control the expression of genes involved in glucose homeostasis, lipid metabolism, inflammation, and cell differentiation. Here, we analyzed the effects of aleglitazar, a dual PPARα and PPARγ agonist with balanced affinity for either subtype, on subacute stroke outcome. Healthy young adult mice were subjected to transient 30 min middle cerebral artery occlusion (MCAo)/reperfusion. Daily treatment with aleglitazar was begun on the day of MCAo and continued until sacrifice. Blood glucose measurements and lipid profile did not differ between mice receiving aleglitazar and mice receiving vehicle after MCAo. Aleglitazar reduced the size of the ischemic lesion as assessed using NeuN immunohistochemistry on day 7. Sensorimotor performance on the rotarod was impaired during the first week after MCAo, an effect that was significantly attenuated by treatment with aleglitazar. Smaller lesion volume in mice treated with aleglitazar was accompanied by a decrease in mRNA transcription of *IL-1β*, *Vcam-1*, and *Icam-1*, suggesting that reduced proinflammatory signaling and reduced vascular inflammation in the ischemic hemisphere contribute to the beneficial effects of aleglitazar during the first week after stroke. Further experiments in primary murine microglia confirmed that aleglitazar reduces key aspects of microglia activation including NO production, release of proinflammatory cytokines, migration, and phagocytosis. In aggregate, a brief course of PPARα/γ agonist aleglitazar initiated post-event affords stroke protection and functional recovery in a model of mild brain ischemia. Our data underscores the theme of delayed injury processes such as neuroinflammation as promising therapeutic targets in stroke.

**Key messages:**

- PPARα/γ agonist aleglitazar improves stroke outcome after transient brain ischemia.
- Aleglitazar attenuates inflammatory responses in post-ischemic brain.
- Aleglitazar reduces microglia migration, phagocytosis, and release of cytokines.
- Beneficial effects of aleglitazar independent of glucose regulation.
- Aleglitazar provides extended window of opportunity for stroke treatment.

## Introduction

Peroxisome proliferator-activated receptors (PPARs) play an important role in regulating the expression of genes associated with glucose homeostasis, lipid metabolism, inflammation, and cell differentiation [1]. While PPARα agonism is primarily associated with hypolipidemic effects, PPARγ plays a critical role in increasing insulin sensitivity of target tissues and promoting adipocyte differentiation [2]. Several reports have appeared affirming neuroprotective and anti-inflammatory effects of PPARγ agonists in rodent middle cerebral artery occlusion (MCAo) models [3–8]. Interestingly, these beneficial effects on stroke outcome must at least be partially independent of glucose homeostasis because they may also be observed in normotensive, normoglycemic animals (e.g., [6]). Although the role of PPARα in ischemic brain injury is less well defined, there is some evidence to indicate that fibrates, agonists of PPARα [9], may also exert protective effects in the ischemic brain [10].

It has recently been hypothesized that dual PPARα/γ agonists may hold special therapeutic promise [1, 11]. Aleglitazar is among the last dual PPARα/γ agonists in clinical development [12]. The high potency and balanced activity on PPARα and PPARγ confer to aleglitazar a quite unique profile relative to other ligands [1]. The current study was designed to test the efficacy of aleglitazar in a mouse model of mild transient focal brain ischemia. Importantly, our investigation combines complex endpoints including histological stroke outcome, sensorimotor assessments, metabolic measures, and a detailed study of the effects of aleglitazar on microglia and on inflammatory signaling in the ischemic brain. To our knowledge, this is also the first report to examine the effects of a dual PPARα/γ agonist in a model of middle cerebral artery occlusion.

## Methods

### Cell culture experiments

Primary neuronal cultures of mouse cerebral cortex were obtained from fetal C57BL/6N mice (E15) essentially as described previously [13, 14]. Briefly, cerebral cortex was dissected, incubated for 15 min in trypsin/EDTA (0.05/0.02% *w*/*v* in phosphate-buffered saline) at 37 °C, rinsed twice with phosphate-buffered saline and once with dissociation medium (modified Eagle’s medium with 10% fetal calf serum, 10 mM HEPES, 44 mM glucose, 100 U/ml penicillin/streptomycin, 2 mM l-glutamine, 0.025 IU/ml insulin), mechanically dissociated with Pasteur pipette in dissociation medium, pelleted by centrifugation (at 225×*g* for 2 min), redissociated in starter medium (Neurobasal medium and supplement B27 [Gibco], 100 U/ml penicillin/streptomycin, 0.5 mM l-glutamine, 25 μM glutamate), and plated in 24-well plates at a density of 3.5 × 10^5^ cells/well. Wells were precoated by incubation with poly-l-lysine (5 μg/ml in phosphate-buffered saline; Biochrom) at 4 °C overnight, then gently rinsed once with phosphate-buffered saline, followed by incubation with collagen medium (modified Eagle’s medium with Earle’s salts, 5% fetal calf serum, 10 mM HEPES, 100 U/ml penicillin/streptomycin, 3% *w*/*v* collagen G; Biochrom) for 1 h at 37 °C. Finally, wells were gently rinsed twice with phosphate-buffered saline. Cultures were fed on in vitro day 4 with cultivating medium (starter medium without glutamate) by replacing 30% of the medium. Experiments were performed after in vitro day 10.

Oxygen–glucose deprivation (OGD) was performed as described previously [13]. Briefly, after removal of medium, cultures were challenged with oxygen–glucose deprivation in a balanced salt solution within a humidified, temperature-controlled (36 ± 0.5 °C) anaerobic chamber (0.3% O_2_; INVIVO_2_ 400, Ruskinn Life Sciences). Neuronal cultures were pretreated with aleglitazar (125 μM) 24 h prior to OGD. Vehicle-treated neurons received 0.25% DMSO in culture medium.

Cultures of primary postnatal murine microglia were obtained from cortex and midbrain of newborn C57BL/6N mice (P0–3) as previously described [15]. Briefly, microglial cells were harvested by gentle shake off and seeded into either 6- or 48-well plates at an initial density, which was respectively 2 × 10^6^ and 2 × 10^5^ cells per well. Cells remained in culture for an additional 24 h before use. The purity of cultures exceeds 98% as previously verified by flow cytometry [16].

All microglia experiments were performed in DMEM (Invitrogen/Gibco, Karlsruhe, Germany) containing 10% fetal calf serum (FCS), 1% penicillin/streptomycin, 1% sodium pyruvate, and 4.5 g/l D-glucose (“complete medium”; all from Biochrom/Merck KGaA, Darmstadt, Germany). Lipopolysaccharide (LPS; *E. coli* 055:B5; Sigma-Aldrich, Saint Louis, MO, USA) was applied at a concentration of 1 μg/ml [15].

### Cell viability assays

Cellular viability was assayed by measuring intracellular reduction of the tetrazolium salt 3-(4,5-dimethylthiazol-2-yl)2,5-diphenyl tetrazolium bromide (MTT) to formazan. The MTT labeling agent (Sigma-Aldrich) was added to the cells at a final concentration of 0.5 mg/ml. The converted dye was solubilized in 10% SDS in 0.01 M HCl and measured at 550 nm with a plate reader (TriStar LB941, Berthold Technologies).

The CytoTox-Glo™ Cytotoxicity Assay kit (Promega) was used according to the manufacturer’s instructions. Aliquots of cell culture medium were collected for analysis of lactate dehydrogenase (LDH) activity as described previously [17].

### Measurement of nitric oxide release

Nitric oxide (NO) production was quantified as nitrite accumulation using the Griess reagent for nitrite (Sigma-Aldrich) as described previously [15]. Briefly, cells were cultured in 48-well plates at a density of 2 × 10^5^ cells/well and treated with aleglitazar (250, 125, 62.5 or 31.3 μM) or vehicle (DMSO) for 48 h. Aliquots of 50 μl cell culture supernatant were incubated with 50 μl Griess’ reagent. Absorption was measured at 550 nm with a microplate spectrophotometer (TriStar LB941, Berthold Technologies, Bad Wildbad, Germany).

### Cytokine measurements

The cytokines TNF-α, IL-6, IL-1β, and CXCL1 were measured by ELISA (TNF-α: eBioscience, San Diego, CA; IL-6 and IL-1β: R&D Systems, Minneapolis, MN; IL-1β and CXCL1 in serum: MSD Mouse ProInflammatory 7-Plex Kit no. N75012B) according to each manufacturer’s instructions.

### Messenger RNA isolation and polymerase chain reactions

Brain tissue was homogenized and total RNA extracted using TRIzol® reagent (Invitrogen). For polymerase chain reaction amplification, we used gene-specific primers (Table 1) and Light Cycler 480 SYBR Green I Master (Roche Diagnostics). Polymerase chain reaction conditions were as follows: preincubation 95 °C, 10 min; 95 °C, 10 s, primer-specific annealing temperature, 10 s, 72 °C, 15 s (45 cycles). Crossing points of amplified products were determined using the Second Derivative Maximum Method (Light Cycler 480 Version 1.5.0, Roche). Quantification of messenger RNA expression was relative to receptor accessory protein 5 (*Reep5*; [18]). Specificity of polymerase chain reaction products was checked using melting curve analysis and electrophoresis on a 1.5% agarose gel.

**Table 1:** Oligonucleotide sequences of primers used in quantitative real-time polymerase chain reaction

Gene	Sense	Antisense
IL-1β	CAA CCA ACA AGT GAT ATT CTC CAT G	GAT CCA CAC TCT CCA GCT GCA
IL-6	GAG GAT ACC ACT CCC AAC AGA CC	AAG TGC ATC ATC GTT GTT CAT ACA
iNOS	GCT CGC TTT GCC ACG GAC GA	AAG GCA GCG GGC ACA TGC AA
eNOS	CAG GAC TGC ACA GGA AAT GTT C	AGC ACA TCA AAG CGG CCA TTT C
Vcam-1	CTC CCG TCA TTG AGG ATA TTG G	CTG GGA GAG ATG TAG ACT TGT AG
Icam-1	CAGTCCGCTGTGCTTTGAGAAC	GCACCGTGAATGTGATCTCCTTG
Kdr	GCA TGG TCT TCT GTG AGG CAA AG	GAG AGT GCC AGG TGA AAT CAA GC
Reep5	CTG ATA GGT TTC GGA TAC CCA G	GAC TCG TGC TTG AGG AAG ATA G
Tnf-α	CAT CTT CTC AAA ATT CGA GTG ACA A	TGG GAG TAG ACA AGG TAC AAC CC
IL-18	GAC TCT TGC GTC AAC TTC AAG G	CTC GAA CAC AGG CTG TCT TTT G
Nfkb	GTC AAC AGA TGG CCC ATA CCT TC	GTC CTG CTG TTA CGG TGC ATA C
Acox1	GAG GAC TAT AAC TTC CTC ACT CG	GAT GAG TTC CAT GAC CCA TCT C
Ehhadh	GCT ATG ATC CGC CTC TGC AAT C	CCT AAT GTA AGG CCA GTG GGA G
Fabp4	GTC TCC AGT GAA AAC TTC GAT G	GTT ATG ATG CTC TTC ACC TTC C
Insr	CAC TCC TAC TGC TAT GGG CTT C	CCT CAA TGA CTG AGC AGT TCT CC

### Western blot

After sacrifice, brains were quickly removed, flash frozen in dry ice-cooled isopentane, and stored until further use. Protein concentration was determined with the Pierce™ BCA Protein Assay Kit (ThermoFisher Scientific Inc.). Equal amounts of protein were loaded on 10% SDS-polyacrylamide gels and blotted onto Immobilon®-FL PVDF membranes (Merck KGaA). Near-infrared fluorescent signals were detected with the Odyssey® CLx Infrared Imaging System (LI-COR, Inc.). Densitometric quantification of band intensity was performed with the Image Studio™ Lite Software (LI-COR). The following primary antibodies were used: rabbit anti-VCAM-1 (no. ab134047, Abcam) 1:4000; rabbit anti-ICAM-1 (no. ab179707, Abcam) 1:1000; rabbit anti-GAPDH (no. 2118, Cell Signaling) 1:1000. The following secondary antibody was used: donkey anti-rabbit IgG (no. 925–32,213, LI-COR) 1:10000.

### Modified Boyden chamber assay

Primary microglia were seeded onto FluoroBlok™ Inserts (8 μm pore size, Corning Incorporated) at a density of 3 × 104 cells/Transwell insert. Both the insert and the well underneath contained cell culture medium supplemented with 250 μM aleglitazar or vehicle (DMSO 0.5%). One hundred micromolar ATP (Sigma-Aldrich) was added to the well below the insert. After 6-h incubation at 37 °C and 5% CO2, the membranes of the inserts were stained with 10 μm CFSE dye (Sigma-Aldrich), fixed with 4% paraformaldehyde (PFA), and counterstained with 2 μm 4′,6′-diamidino-2-phenylindole (DAPI; Sigma-Aldrich). Migrated cells below the FluoroBlok membranes were visualized using an inverted fluorescence microscope at × 200 magnification (Leica DMI3000).

### Microglial phagocytosis

Phagocytosis of bacterial particles was assessed using the pHrodo™ Red *E. coli* Bioparticles® Conjugate for phagocytosis according to the manufacturer’s protocol (Thermo Fisher Scientific). Primary microglia were pretreated with aleglitazar (125 μM) or vehicle (DMSO) for 24 h. Excitation wavelength was 550 nm and the fluorescence of the pHrodo™ Red conjugate was collected at 620 nm using a plate reader (TriStar LB941, Berthold Technologies).

### Animals

All animal studies and experimental procedures were approved by the necessary official committees and conducted in compliance with the requirements set out in the European Communities Council Directive of November 24, 1986 (86/609/EEC) and the ARRIVE guidelines [19]. Male 129S6/SvEv mice raised under specific-pathogen-free (SPF) conditions were provided by the Forschungseinrichtungen für Experimentelle Medizin (FEM) of the Charité Universitätsmedizin Berlin. Young adult mice (~ 10 ± 1 weeks old) weighing between 24 and 30 g were randomly used for experiments. Animals were maintained in a temperature (22 °C ± 2 °C) and humidity (55% ± 10%)-controlled environment with a 12:12-h light-dark cycle and ad libitum access to food and water. Aleglitazar was dissolved in a mixture of peanut oil, 0.9% saline, and ethanol (25:37.5:37:5, *v*/*v*), and was administered i.p.

### Induction of cerebral ischemia

The standard operating procedures “Middle cerebral artery occlusion in the mouse” published by Dirnagl and the members of the MCAO-SOP group were followed [20].

Briefly, mice were anesthetized for induction with 1.5% isoflurane and maintained in 1.0% isoflurane in 69% N_2_O and 30% O_2_ using a vaporizer. Left MCAo was induced with an 8.0 nylon monofilament coated with a silicone resin/hardener mixture (Xantopren M Mucosa and Activator NF Optosil Xantopren, Haereus Kulzer). The filament was introduced into the internal carotid artery up to the anterior cerebral artery. Thereby, the middle cerebral artery and anterior choroidal arteries were occluded. The filament was removed after 30 min to allow reperfusion.

**Fig. 1:**
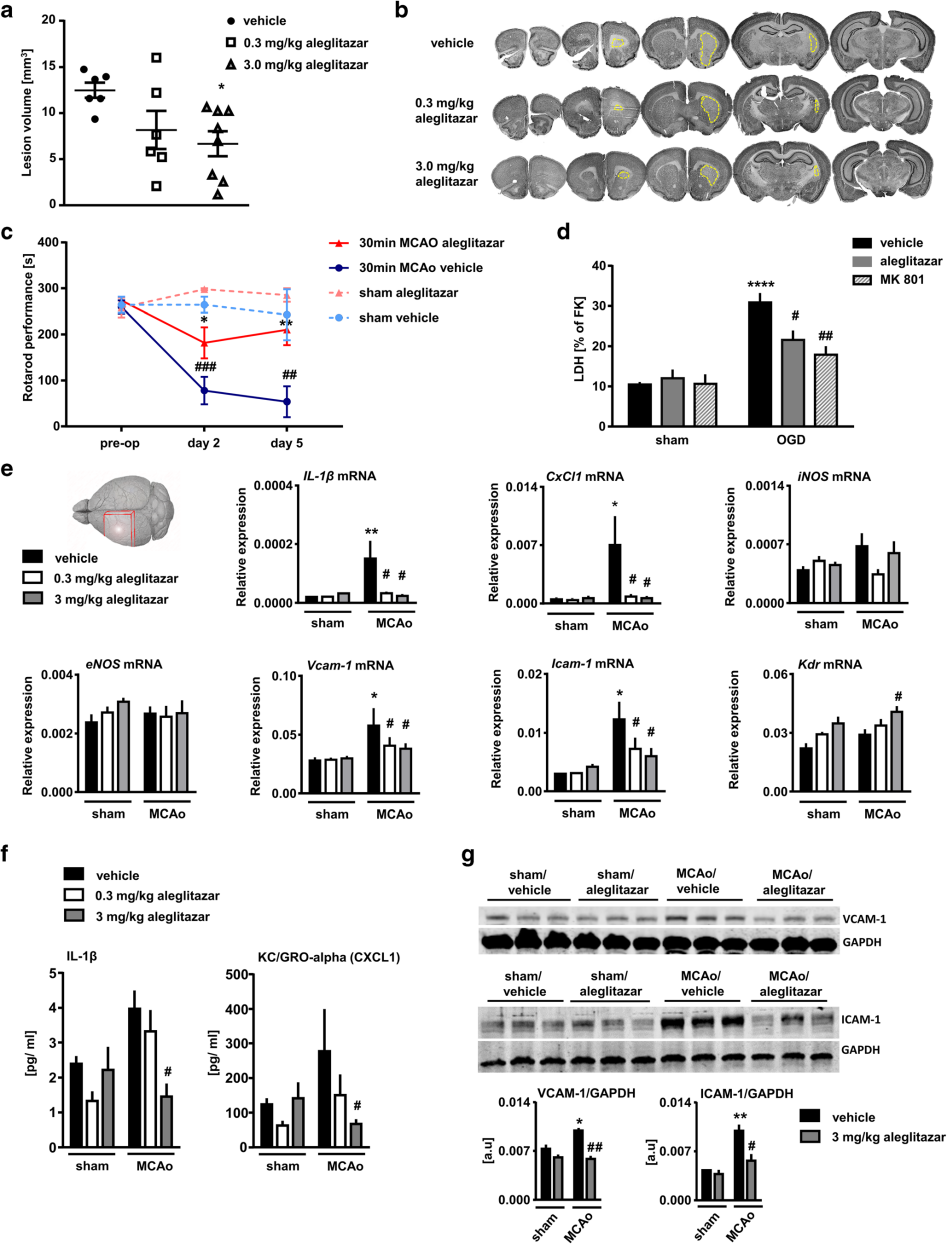
Aleglitazar reduces stroke damage and expression of inflammatory molecules after mild transient brain ischemia. Treatment with aleglitazar was begun on the day on which brain ischemia was induced. Daily treatment with aleglitazar was continued until sacrifice (**a**, **b**). Infarct size at 7 days after 30 min MCAo/reperfusion was assessed using NeuN-stained coronal brain sections. *N* = 6–8 mice per group. One-way ANOVA followed by Tukey’s multiple comparison test. *p < 0.05 versus vehicle-treated control mice. **c** Aleglitazar improves sensorimotor outcome after 30 min MCAo/reperfusion. *N* = 3–7 mice per group. Two-way ANOVA followed by Tukey’s multiple comparison test within each time point. *p < 0.05 and **p < 0.01 aleglitazar versus vehicle, ^##^*p* < 0.01 and ^###^*p* < 0.001 MCAo versus sham. **d** Aleglitazar provides neuroprotection against oxygen–glucose deprivation (OGD). Primary cortical neurons were pretreated with aleglitazar or vehicle for 24 h. Neuronal injury was assessed by measuring lactate dehydrogenase (LDH) release into the cell culture medium 24 h after OGD. MK-801 served as a positive control. *N* = 5 independent cultures per condition. Two-way ANOVA followed by Tukey’s multiple comparison test. ****p < 0.0001 relative to sham cultures; ^#^*p* < 0.05, ^##^*p* < 0.01 for the effect of treatment with aleglitazar within the OGD condition. **e** Expression of inflammatory and angiogenesis-related genes in the ipsilateral, i.e., ischemic MCA territory at 7 days. Relative mRNA expression is reported as the value normalized to receptor accessory protein 5 (*Reep5*). *N* = 4–5 mice per group. Two-way ANOVA followed by Tukey’s multiple comparison test. *p < 0.05 relative to sham. ^#^*p* < 0.05 relative to vehicle-treated MCAo animals. **f** IL-1β and KC/GRO-alpha (CXCL1) concentrations were also measured in serum at 7 days. *N* = 4–5 mice per group. Two-way ANOVA followed by Tukey’s multiple comparison test. ^#^*p* < 0.05 relative to vehicle-treated MCAo animals. **g** VCAM-1 and ICAM-1 Western blots were performed at 7 days. *N* = 3 mice per group. Two-way ANOVA followed by Tukey’s multiple comparison test. *p < 0.05 and **p < 0.01 relative to sham. ^#^*p* < 0.05 and ^##^*p* < 0.01 relative to vehicle-treated MCAo animals

Altogether, three experiments were conducted. The first experiment was performed to study gene expression in brain and liver (Figs. 1e and 3a). Twenty-seven mice (*n* = 5 vehicle sham; *n* = 5 0.3 mg/kg aleglitazar sham; n = 5 3.0 mg/kg aleglitazar sham; *n* = 4 vehicle MCAo; *n* = 4 0.3 mg/kg aleglitazar MCAo; *n* = 4 3.0 mg/kg aleglitazar MCAo) were used. None of these animals died.

The second experiment was performed to study the effects of aleglitazar on histological stroke outcome (Fig. 1a, b). Twenty-four mice were entered into this experiment (*n* = 7 vehicle MCAo; *n* = 8 0.3 mg/kg aleglitazar MCAo; *n* = 9 3.0 mg/kg aleglitazar MCAo). Two mice from the 0.3 mg/kg aleglitazar MCAo group died shortly after the stroke procedure. Moreover, for technical reasons (i.e., no histological evidence of ischemic brain damage), two further animals (*n* = 1 mouse from the vehicle MCAo group; *n* = 1 mouse from the 3.0 mg/kg aleglitazar MCAo group) had to be excluded.

The third experiment was conducted to study post-stroke sensorimotor performance (Fig. 1c). Moreover, glucose and lipid measurements were performed in these animals (Fig. 3b–g). Twenty-two mice were used (*n* = 4 vehicle sham; *n* = 4 3.0 mg/kg aleglitazar sham; *n* = 6 vehicle MCAo; *n* = 8 3.0 mg/kg aleglitazar MCAo). Two mice died after the MCAo procedure (*n* = 1 mouse from the vehicle MCAo group; *n* = 1 mouse from the 3.0 mg/kg aleglitazar MCAo group). Two sham-operated animals (*n* = 1 mouse from the vehicle sham group; *n* = 1 mouse from the 3.0 mg/kg aleglitazar sham group) displayed very poor baseline rotorod performance (i.e., even before the sham procedure had been performed). These two animals were excluded from the rotarod experiment.

### Glucose measurements

Blood from the tail vein was used to measure blood glucose levels using a blood glucose monitoring system (FreeStyle Precision, Abbott, Wiesbaden, Germany).

### Serum measurements

Serum was obtained from blood collected after decapitation. Serum cholesterol (Abcam no. 65390) and triglycerides (Abcam no. 65336) were quantified as per manufacturer’s instructions.

### Immunohistochemistry and determination of infarct size

All procedures have been described in detail previously [21, 22]. Briefly, brains were perfusion fixed with 4% paraformaldehyde and cut into 40-μm thick coronal sections. Immunohistochemistry followed the peroxidase method with a biotinylated secondary antibody (Jackson ImmunoResearch Laboratories, West Grove, Pennsylvania), ABC Elite reagent (Vector Laboratories, Burlingame, California), and diaminobenzidine (Sigma) as chromogen. The following primary antibodies were used: mouse anti-NeuN (MAB377B, Chemicon) 1:100 and rabbit anti-Iba1 (019-19741, Wako) 1:500. Lesion volume was quantified as described elsewhere [23]. The number of Iba1+ activated microglia per volume was assessed using StereoInvestigator® software (MicroBrightfield) as described previously [13, 16]. Ischemic lesion size was measured by computer-assisted volumetry of serial 40-μm-thick NeuN-stained coronal brain sections (~ 2 mm apart) as described previously [24].

### Rotarod performance

The Rotarod (TSE Sytems) was used to gauge general fitness and motor coordination as described in detail elsewhere [13]. Briefly, mice were placed on an accelerating rotating beam (acceleration from 2 rpm to 40 rpm within 5 min) and a stop-clock was started. The latency for each animal to fall off the rotarod onto the sensing platform below was recorded. Each mouse performed three trials.

### Statistics

Experiments were carried out in a blinded fashion. Data are presented as mean ± SEM. Unless otherwise indicated, groups were compared by ANOVA with level of significance set at 0.05 and two-tailed *p* values using GraphPad Prism 6 (GraphPad software).

## Results

### Aleglitazar exerts beneficial effects on structural and functional outcomes of mild brain ischemia

We studied the effects of daily aleglitazar on histological stroke outcome in mice subjected to 30 min MCAo/reperfusion at 7 days post-stroke (Fig. 1a, b). While the effects of the lower dose of aleglitazar (0.3 mg/kg) on brain lesion volume failed to reach significance, the higher dose (3 mg/kg) yielded a statistically significant and biologically meaningful decrease of ~ 50% in infarct size (Fig. 1a, b). Histological stroke protection associated with the higher dose of aleglitazar was also reflected at the functional level in the form of improved sensorimotor performance on the rotarod at 2 and 5 days post-MCAo (Fig. 1c).

Next, we tested cultures of primary cortical neurons and applied aleglitazar 24 h before oxygen–glucose deprivation (OGD). Aleglitazar provided robust protection against cell death as quantified by LDH release after 180 min OGD (Fig. 1d). Glutamate antagonist MK-801 (10 μm) served as a positive control.

Subsequently, we investigated the expression of key inflammatory and angiogenesis-related genes in the ischemic hemisphere at 7 days (Fig. 1e). In particular, IL-1 has been shown to be a major contributor to ischemic brain damage [25]. Aleglitazar strongly repressed *IL-1β* mRNA and chemokine (C-X-C motif) ligand 1 (*CxCl1*) mRNA at both concentrations investigated. The adhesion molecules VCAM-1 and ICAM-1 are induced in endothelium during inflammation and play a pivotal role in mediating leukocyte adhesion [26–28]. Treatment with aleglitazar attenuated the stroke-induced increase in mRNA expression of both of these markers of vascular inflammation. By contrast, we observed a significant upregulation of *Kdr* (VEGF receptor 2) in the ischemic hemisphere of mice on 3 mg/kg aleglitazar, which would be consistent with the idea that aleglitazar may promote post-MCAo angiogenesis [13, 29].

To further bolster these mRNA results, we measured IL-1β and KC/GRO-alpha (CXCL1) concentrations in serum. Relative to vehicle-treated MCAo mice, MCAo mice treated with 3.0 mg/kg aleglitazar showed significantly reduced concentrations of these two proinflammatory cytokines.

Finally, we studied protein expression of VCAM-1 and ICAM-1 in brain (Fig. 1g). Both VCAM-1 and ICAM-1 were increased in the ischemic hemisphere, an effect which was again counteracted by aleglitazar.

### Aleglitazar reduces microglia activation

**Fig. 2:**
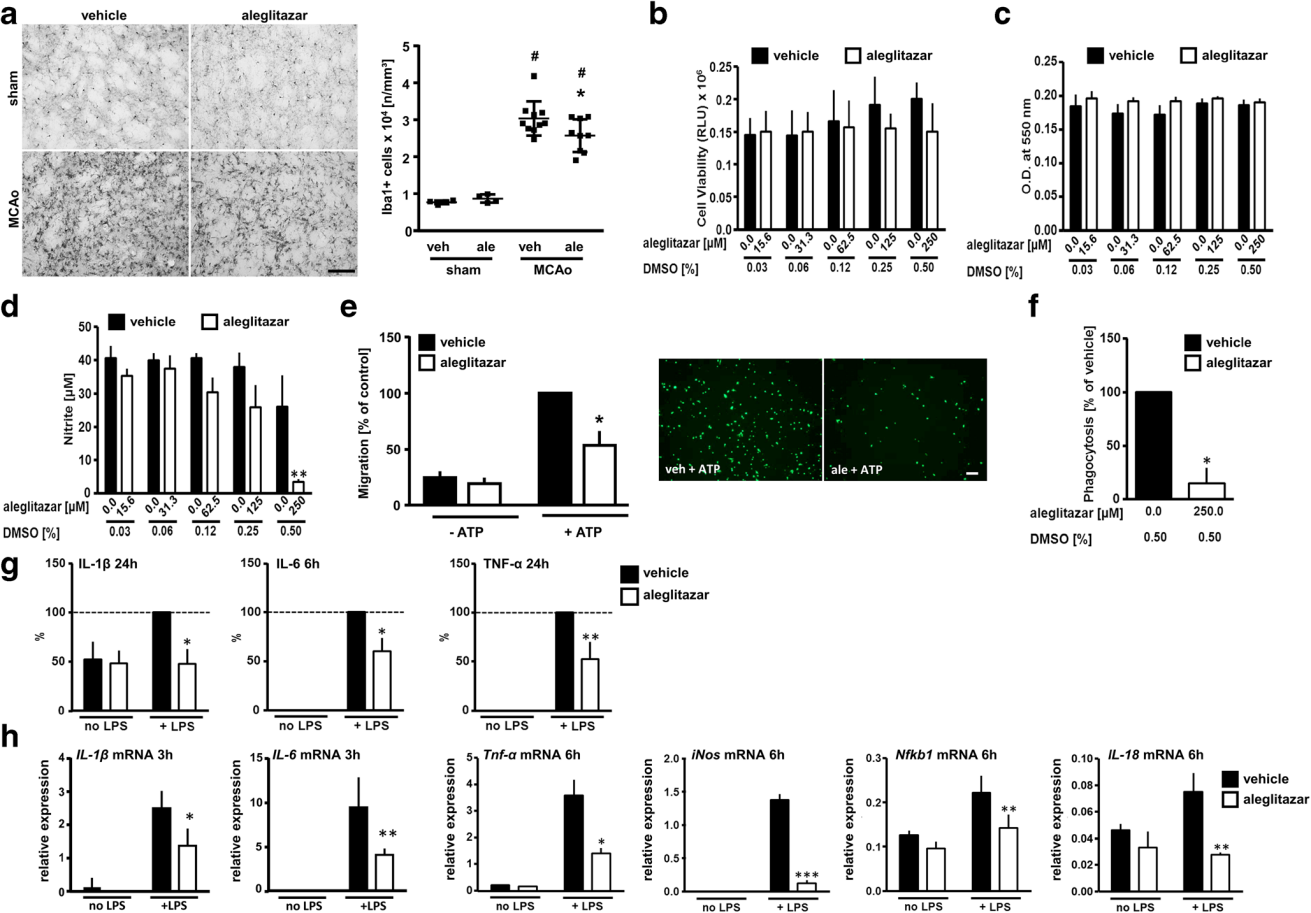
Aleglitazar and microglial behavior. **a** Density of Iba1-immunoreactive microglia/macrophages in the ischemic lesion at 7 days after MCAo/reperfusion. The corresponding area of sham-operated mice served as control tissue. Treatment with daily aleglitazar (3 mg/kg) was begun on the day of MCAo. *N* = 4–10 mice per group. Two-way ANOVA followed by Tukey’s multiple comparison test. ^#^*p* < 0.01 MCAo vs. sham within each treatment condition. *p < 0.05 relative to vehicle-treated MCAo animals. **b**, **c** Aleglitazar was applied for 48 h. Viability of primary postnatal mouse microglia was assessed using a luminescent cytotoxicity assay (**b**) and the MTT dye assay (**c**). At the concentrations employed, aleglitazar did not affect cell viability. **d** LPS was applied at a concentration of 1 μg/ml for 48 h. NO release from microglia was quantified as nitrite accumulation. **e** Cell migration in a modified Boyden chamber chemotaxis assay was measured over a time period of 4 h. Aleglitazar significantly reduced migration stimulated by ATP. Scale bar 200 μm. **f** Phagocytosis was measured based on the uptake of bacterial particles conjugated to pH-sensitive dye over a span of 2 h. **g** Release of IL-1β, IL-6, and TNF-α was measured after 6 h or 24 h incubation with LPS (1 μg/ml). **h** mRNA expression of key genes associated with classical microglia activation after 3 h or 6 h incubation with LPS. Data in **e**–**g** were normalized due to variation between different experiments. Two-way ANOVA followed by Tukey’s multiple comparison test (**b**–**e**, **g**, **h**); unpaired Student’s *t* test (**f**). *p < 0.05, **p < 0.01, ***p < 0.005 relative to vehicle (DMSO), minimum of three independent measurements per data point. RLU relative light units

Neuroinflammation is a powerful determinant of stroke outcome [30]. We therefore measured the density of Iba1-immunoreactive-activated microglia/macrophages in the ischemic lesion at 7 days after MCAo/reperfusion and in the corresponding brain area of sham-operated mice (Fig. 2a). The density of Iba1-immunoreactive cells was strongly increased in the ischemic brain. Relative to vehicle-treated MCAo mice, animals treated with aleglitazar displayed a significant reduction in Iba1+ cells in the ischemic MCA territory (Fig. 2a).

We then investigated the effects of aleglitazar on microglia behaviors in vitro. Aleglitazar did not decrease microglia viability across the wide range of concentrations investigated (Fig. 2b, c). Generation of nitric oxide is a hallmark of LPS-induced microglia activation [31, 32]. At a concentration of 250 μM, aleglitazar robustly suppressed NO release (Fig. 2d). While aleglitazar did not affect constitutive microglia migration in the absence of ATP, ATP-induced chemotaxis was reduced by ~ 68% in the presence of aleglitazar (Fig. 2e). Similarly, phagocytosis of bacterial particles was greatly reduced in the presence of aleglitazar (Fig. 2f). Release of IL-1β, IL-6, and TNF-α from LPS-activated microglia was quantified at the protein level by ELISA (Fig. 2g). Pretreatment with aleglitazar for 24 h significantly reduced release of IL-1β, IL-6, and TNF-α into the culture medium (Fig. 2g). Finally, mRNA expression of *IL-1β*, *IL-6*, *Tnf-α*, *iNos*, *Nfkb1*, and *IL-18*, key genes associated with microglia activation, was significantly reduced by treatment with aleglitazar.

### Metabolic effects of aleglitazar

**Fig. 3:**
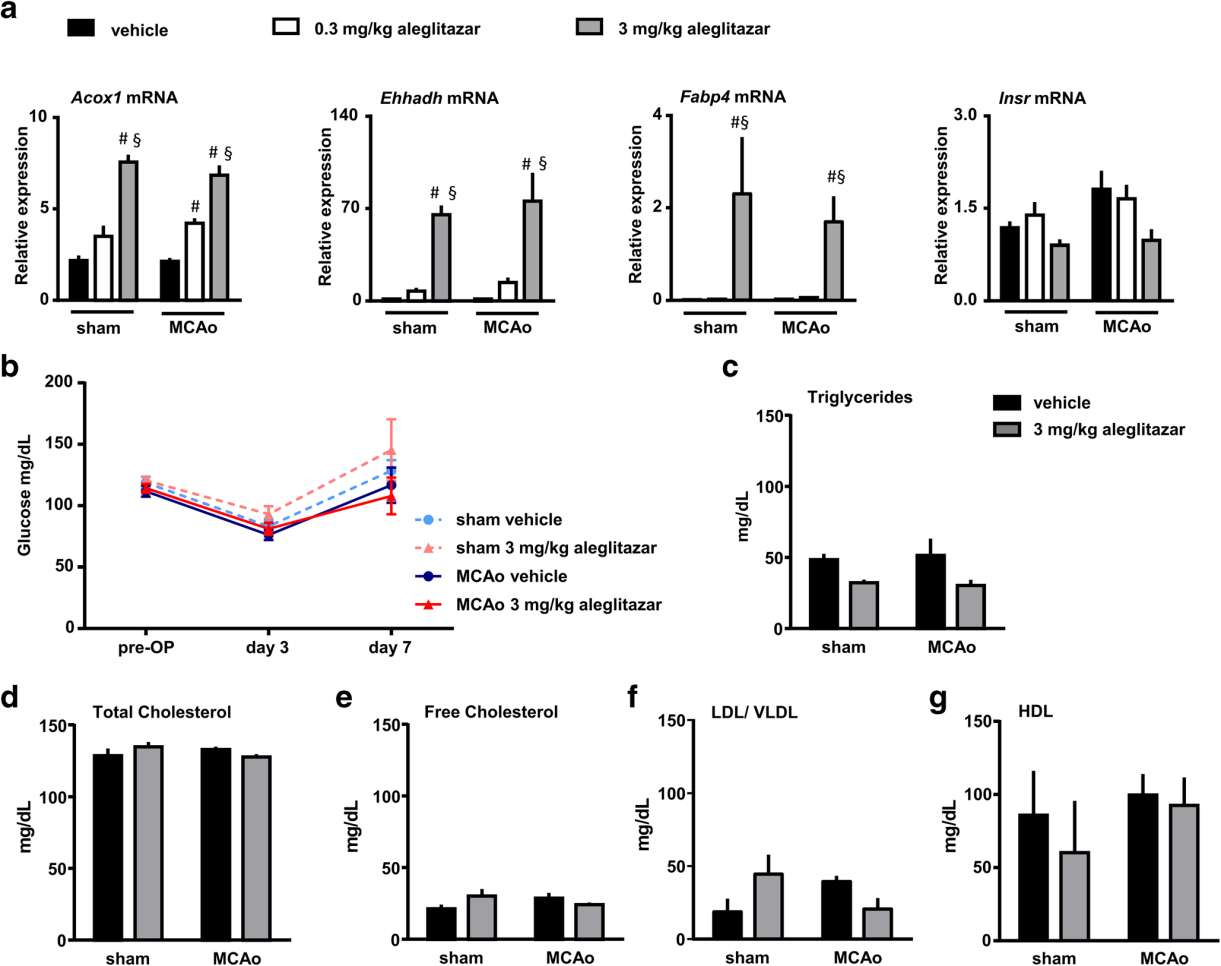
Metabolic effects of aleglitazar. **a** Relative mRNA expression of key molecules involved in hepatic lipid and glucose metabolism. Mice received aleglitazar or vehicle for 7 consecutive days after 30 min MCAo/reperfusion or sham surgery and were euthanized on day 7. MRNA expression is reported as the value normalized to receptor accessory protein 5 (*Reep5*). *N* = 4–5 mice per group. Two-way ANOVA followed by Tukey’s multiple comparison test. ^#^*p* < 0.05 relative to vehicle. ^§^*p* < 0.05 relative to the lower aleglitazar concentration. *Acox1*: acyl-coenzyme A oxidase 1, *Ehhadh*: enoyl-CoA hydratase, *Fabp4*: fatty acid bing protein 4, *Insr*: insulin receptor. **b** Blood glucose measurements were performed before and after surgery. *N* = 4–7 mice per group. Two-way ANOVA followed by Tukey’s multiple comparison test within each time point. **c**–**g** The effects of aleglitazar on the lipid profile were evaluated on day 7 after 30 min MCAo/reperfusion or sham surgery. *N* = 3–7 mice per group. Two-way ANOVA followed by Tukey’s multiple comparison test

To study the metabolic effects of aleglitazar, we studied mRNA expression of acyl-coenzyme A oxidase 1 (*Acox1*), enoyl-coenzyme A hydratase (*Ehhadh*), fatty acid binding protein (*Fabp4*), and the insulin receptor (*Insr*) in the liver at 7 days after MCAo/reperfusion or sham surgery (Fig. 3a). While MCAo did not affect mRNA transcription of any of the genes investigated, aleglitazar dose-dependently induced *Acox1*, *Ehhadh*, and *Fabp4* mRNA levels.

Blood glucose measurements were performed before and after the MCAo procedure (Fig. 3b). Glucose levels did not differ significantly between experimental groups at any of the time points investigated. Similarly, hepatic mRNA expression of the insulin receptor did not differ across groups (Fig. 3a). Finally, we did not detect significant differences in the lipid profile between mice receiving aleglitazar and mice receiving vehicle (Fig. 3c–g).

## Discussion

As transcription factors regulating a wide array of genes implicated in metabolism, proliferation, inflammation, and cell differentiation, peroxisome proliferator-activated receptors (PPARs) are being investigated as promising therapeutic targets in the metabolic syndrome, cardiovascular disorders, and stroke [33]. What sets aleglitazar apart from other compounds is that it is a dual PPARα/γ agonist with balanced affinity for both subtypes [1]. Interestingly, an analysis of gene transcript data from human hepatocytes revealed that the molecular effects of aleglitazar are distinct from those of tesaglitazar (another dual PPARα/γ agonist) or the combination of pioglitazone/fenofibrate [34].

Clinical development of aleglitazar was halted in mid-2013 after the AleCardio trial was stopped upon the recommendation of an independent data and safety monitoring board after a median follow-up of 104 weeks [35]. This phase 3 trial had set out to determine whether long-term treatment with aleglitazar in addition to standard medical therapy would reduce cardiovascular morbidity and mortality among patients with type 2 diabetes mellitus and a recent acute coronary syndrome. The trial was terminated due to a lack of efficacy of chronic aleglitazar treatment on cardiovascular outcomes in conjunction with increased rates of safety end points, especially gastrointestinal hemorrhages and renal dysfunction [35].

The present study was conceived and begun before the AleCardio trial was stopped and Roche discontinued clinical development of aleglitazar. Our experimental study was designed to assess the effects of a 1-week course of aleglitazar on complex outcomes of focal stroke. Using a well-established mouse model of transient mild brain ischemia, we investigated histological, molecular, neurobehavioral, and metabolic endpoints at 7 days post-event. The 7-day time point affords an excellent window on the subacute stage of brain ischemia because it reflects the intertwined effects of multiple dynamic processes such as neuronal loss, neuroinflammation, and gliosis. In parallel, we performed cell culture experiments to study, in a single cell type, the direct effects of aleglitazar on the survival of primary neurons following OGD and on microglia acutely activated by stimulation with LPS. Our study yielded the following key results: (1) Aleglitazar conferred neuroprotection against 180 min OGD. Moreover, brief treatment with aleglitazar significantly reduced subacute infarct size and improved sensorimotor performance following 30-min MCAo/reperfusion. (2) Reduced lesion volume was accompanied by a molecular pattern of reduced proinflammatory signaling and enhanced vascular inflammation in the ischemic hemisphere. Experiments in primary murine microglia further revealed that aleglitazar attenuates key microglial inflammatory responses such as migration, phagocytosis, and the acute release of proinflammatory cytokines. (3) The beneficial effects of short-term aleglitazar on stroke outcome were observed in otherwise healthy (i.e., normoglycemic) young adult mice. In other words, aleglitazar did not exert significant effects on blood glucose levels or lipid profile across the experimental groups investigated here over the span of 1 week.

The mechanisms underlying neuroprotection by aleglitazar may include several pathways. Both neurons and microglia express PPARα and PPARγ [36–40]. Direct neuroprotective effects of pretreatment with PPARγ agonist rosiglitazone on OGD-induced injury have been demonstrated in primary mouse neurons subjected to OGD [41]. Our results suggest that, besides these potential direct protective effects of aleglitazar on neurons, reduced proinflammatory signaling in the post-ischemic brain may play a particularly critical role in mitigating and limiting injury. We found that aleglitazar significantly reduces the release of proinflammatory cytokines by LPS-activated microglia in vitro. Aleglitazar also decreases *IL-1β* mRNA transcription in the ischemic hemisphere. Moreover, MCAo mice treated with 3.0 mg/kg aleglitazar showed significantly reduced concentrations of circulating IL-1β in serum. Taken together, these observations are meaningful because strong experimental evidence from numerous reports supports the notion that IL-1β is a major driver of MCAo-induced brain damage (e.g. [25, 42, 43]. Our finding of reduced phagocytic activity of cultured microglia treated with aleglitazar is equally relevant in the context of stroke. “Phagoptosis,” i.e., phagocytosis of cells exposed to sublethal insults, is a relatively new concept in neuroscience (e.g., [44]). Inhibition of phagocytosis has previously been shown to improve stroke recovery by preventing delayed neuronal death, even though the initial infarct was not affected [45]. In our model of mild transient brain ischemia, neuronal loss is most pronounced in the population of striatal medium spiny projection neurons and typically occurs in a delayed fashion over a period of several days [46, 47]. The fact that, in the experiments reported here, daily treatment with aleglitazar was only begun after the MCAo procedure had been performed points to a potential new and extended window of opportunity for stroke treatment.

One potential limitation of the current report is that we did not measure aleglitazar concentrations in the brain. To our knowledge, it has not yet been determined to which extent aleglitazar crosses the intact blood-brain barrier. However, generally speaking, penetration into the brain is substantially facilitated by disruption of the blood-brain border, a core characteristic of cerebral ischemia (e.g., [48]). The dose of aleglitazar used here is in line with doses investigated in mice in earlier studies (e.g., [49, 50]). Also, since food intake may be unreliable in the first few days after MCAo and mice tend to lose a considerable amount of weight (e.g., [51]), aleglitazar was administered intraperitoneally. On balance, the pattern of results obtained for mRNA transcription in both the ischemic brain and the liver demonstrates the biological activity of aleglitazar in the experiments reported herein, and, in particular, in the post-ischemic brain.

Sensorimotor performance was assessed using the rotarod, one of the most frequently used tests of motor function after proximal MCAo in the mouse [52]. Using our model of 30 min MCAo/reperfusion, we have previously established that the rotarod is a useful and sensitive tool in the evaluation of motor deficits, especially during the first week after mild brain ischemia [53]. On day 5 after MCAo, strong beneficial effects of aleglitazar on rotarod performance were apparent, lending further credence to the translational value and clinical relevance of our findings [52, 54].

To summarize, our report indicates that a brief 1-week course of PPARα/γ agonist aleglitazar initiated post-event affords stroke protection/recovery in a model of mild brain ischemia. Our data underscores the theme of delayed injury processes such as selective neuronal degeneration, phagoptosis, and neuroinflammation after stroke. Short-term treatment with aleglitazar may even hold promise for clinical stroke therapy.
